# Effect of the Single and Combined Use of Curcumin and Piperine on Growth Performance, Intestinal Barrier Function, and Antioxidant Capacity of Weaned Wuzhishan Piglets

**DOI:** 10.3389/fvets.2020.00418

**Published:** 2020-07-31

**Authors:** Liguang Shi, Wenjuan Xun, Weiqi Peng, Haichao Hu, Ting Cao, Guanyu Hou

**Affiliations:** ^1^Tropical Crops Genetic Resources Institute, Chinese Academy of Tropical Agricultural Sciences, Haikou, China; ^2^College of Animal Sciences and Technology, Hainan University, Haikou, China

**Keywords:** curcumin, piperine, growth performance, intestinal permeability, weaned piglet, antioxidant capacity

## Abstract

This study was conducted to evaluate effects of the single and combined use of curcumin (CUR) and piperine (PIP) on performance, intestinal barrier function, and antioxidant capacity of weaned piglets. A total of 50 Wuzhishan piglets weaned at 35 days of age were randomly assigned to five groups receiving a corn–soybean basal diet (CON), the basal diet supplemented with 50 mg/kg piperine, 200 mg/kg curcumin (low-CUR), 200 mg/kg curcumin + 50 mg/kg piperine (PIP + CUR), and 300 mg/kg curcumin (high-CUR), respectively. The results showed that the feed/gain ratio (F/G) and plasma d-lactate and diamine oxidase activity (DAO) of the CUR + PIP and high-CUR groups were lower than those of the CON group (all *P* < 0.05), while the jejunum and ileum villus height, the villus height/crypt depth ratio, and the messenger RNA (mRNA) expression levels of *occludin, claudin-1*, and *zonula occluden-1* in jejunal and ileal mucosa were higher in the CUR + PIP and high-CUR groups than in the CON group (all *P* < 0.05). Moreover, the piglets in the CUR + PIP and high-CUR groups had higher serum and intestinal mucosa activity of superoxide dismutase and glutathione peroxidase and lower malonaldehyde concentration than piglets in the CON group (all *P* < 0.05). The above parameters were not significantly different between the CUR + PIP and high-CUR groups (*P* > 0.05). In conclusion, the combination of CUR and PIP seemed to be as advantageous as high-CUR to piglets, but it was more effective than the single use of CUR and PIP. These data indicated that the basal diet supplemented with CUR + PIP or high-CUR could improve the intestinal permeability and suppress oxidative stress of weaned Wuzhishan piglets.

## Introduction

After weaning, piglets often suffer from intestinal barrier dysfunction, which contributes to severe diarrhea and decreased performance in piglets (1–3). Antibiotics have been widely used as animal feed additives for many years because of their efficiency in increasing the growth rate, improving feed utilization, and reducing mortality (4). However, their continuous use may lead to the emergence of drug resistance and antibiotic residues in poultry products (5), harming human health and the environment (6). As a result, some countries have banned the addition of antibiotics to livestock as growth promoters (7, 8).

In the past two decades, a large number of materials have been investigated as alternatives to antibiotics added in piglet diets (9). Among them, plant extracts are proven to be useful in relieving the post-weaning syndrome (10). Curcumin, an active natural polyphenol derived from the curry spice turmeric, has been widely used as medicine, dietary additives, and coloring agents (11). It exhibits biological activities as diverse as antioxidant (12), antiviral (13), anticancer (14), antiproliferation (15), antidiabetic (16), and anti-inflammatory properties (17). Particularly, the protective effects of CUR on the intestinal mucosa barrier were also repeatedly demonstrated in rat (18, 19), duck (20), and human intestinal epithelial cells (21, 22). Our previous experiments have also shown that a basal diet supplemented with 300 mg/kg CUR could improve the integrity and morphology of the intestinal mucosal barrier as well as the immunity of weaned pigs challenged with enterotoxigenic *Escherichia coli* (23).

In spite of an extensive range of pharmacological potentials, the CUR has limited bioavailability when administered orally, which can be explained by its poor absorption, low stability, and fast metabolism and excretion from the body (24, 25). To increase its bioavailability, several attempts have been made. Piperine, a bioactive alkaloid in pepper, has been suggested to inhibit the hepatic and intestinal glucuronidation and improve the bioavailability of CUR (26–28). The combined use of CUR and PIP has been reported to attenuate inflammation (29–31). The addition of PIP to CUR-containing formulations increases intestinal and plasma concentrations of CUR and thus enhances its inflammation-preventing activities (32). In this paper, we hypothesized that the combined use of CUR and PIP might be more effective than the single use of CUR and PIP in preventing diarrhea and poor performance induced by weaning in pigs.

Wuzhishan pig is a miniature pig breed originating from Hainan Island of China. In the previous study, we found that weaning at the 35th day after birth was more beneficial to the intestinal barrier function of Wuzhishan piglets than weaning at the 21st day after birth (33). Therefore, the purpose of this study was to determine the effects of single and combined use of CUR and PIP on growth performance, intestinal barrier function, and antioxidant capacity of 35-day-old weaned Wuzhishan piglets.

## Methods and Methods

### Materials

Curcumin (90.0%) was purchased from Shi jiazhuang Lv Chuan Bio Technology Co., Ltd (Hebei, China). Piperine was purchased from Shanghai Macklin Biochemical Co., Ltd. (Shanghai China). The determination kits for antioxidant indices, including superoxide dismutase (SOD), glutathione peroxidase (GSH-Px), total antioxidant capacity (T-AOC) and malonaldehyde (MDA), plasma d-lactate, and diamine oxidase (DAO) were all purchased from the Jiancheng Bioengineering Institute of Nanjing (Jiangsu, China). RNAiso Plus and PrimeScript™ RT Reagent Kit were purchased from TakaRa Biotechnology Inc. (Dalian, China).

### Animal, Diets, and Experimental Design

A total of 50 Wuzhishan piglets weaned at 35 days of age were randomly allocated to one of five treatments (*n* = 10) for 21 days, with the initial average body weight of 3.54 ± 0.28 kg. The five treatments were CON, CON + 50 mg/kg PIP, CON + 200 mg/kg curcumin (low-CUR), CON + 200 mg/kg curcumin + 50 mg/kg piperine (PIP + CUR), and CON + 300 mg/kg curcumin (high-CUR). Diets were formulated to meet the nutrient requirements suggested by the Wuzhishan pig breeding technology discipline (Table 1). All the piglets were housed in individual pens with room temperature maintained at 25–27°C.

**Table 1:** Composition and nutrient levels of the basal diet [dry matter (DM) basis] (%).

**Ingredients**	**Content**	**Nutrient levels^b^**	**Content**
Corn	64.00	Digestible energy(MJ/kg)	13.17
Soybean meal	22.80	CP (%)	17.94
Wheat bran	5.00	Ca (%)	0.80
Fish meal	2.00	P (%)	0.60
Whey powder	3.00	Lys (%)	0.86
Limestone	1.10	Met	0.32
CaHPO_4_	0.80	Met + Cys (%)	0.65
NaCl	0.30		
Premix^a^	1.00		
Total	100.00		

a *provided per kilogram of diet: vitamin A, 3,200 IU; vitamin VD_3_, 480 IU; vitamin E, 25 IU; vitamin K_3_, 0.5 mg; vitamin B_1_, 10 mg; vitamin B_2_, 4 mg; vitamin B_12_, 0.03 mg; folic acid, 0.3 mg; nicotinic, 22 mg; pantothenate, 14 mg; biotin, 0.10 mg; chorine, 830 mg; Cu, 15.6 mg; Fe, 100 mg; Mn, 40 mg; Zn, 106 mg; Se, 0.3 mg; I, 0.2 mg*.

b *digestible energy was a calculated value, while the other nutrient levels were measured values*.

During the 21-day experiment period, piglets were allowed *ad libitum* to designated diet and water. The piglets were individually weighed at the beginning and end of the trial (days 0 and 21), and feed consumption for each pig was recorded daily. Average daily gain (ADG), average daily feed intake (ADFI), and F/G ratio were calculated.

### Sample Collection

On day 21, six piglets subjected to each treatment were randomly selected, and a 5-ml blood sample was harvested from the jugular vein of each piglet into a tube with anticoagulant or without anticoagulant. After 20 min standing at room temperature, plasma or serum was obtained, which were centrifuged at 3,000 *g* for 15 min at 4°C and then stored at −20°C for assays. Plasma samples were used for d-lactate and DAO detection, and serum samples were used for the analysis of antioxidant variables.

After blood sampling, the piglets were sacrificed by injection of sodium pentobarbital solution (50 mg/kg of body weight). The middle sections of the jejunum and ileum were isolated aseptically, flushed with physiological saline, and fixed in 4% paraformaldehyde for 24 h for subsequent histological assays. After mucosa samples from the jejunum and ileum were scraped with a razor, they were frozen in liquid nitrogen immediately and stored at −80°C for further assays.

### Plasma D-Lactate and DAO

The levels of d-lactate and DAO in the plasma were detected by using porcine d-lactic acid ELISA kit and DAO assay kit, respectively, according to the manufacturer's instructions (Jiancheng Bioengineering Institute of Nanjing, Nanjing, China).

### Intestinal Morphology Analysis

The intestinal mucosa morphology including the villus height, villus width, and crypt depth were measured as previously described (23). Briefly, the paraformaldehyde-fixed tissue was embedded in paraffin according to standard procedures. Five-micrometer-thick sections were installed on glass slides. After deparaffinization, the slides were stained with hematoxylin and eosin. The villus height, villus width, and crypt depth were measured by using the Axioskop-2 microscope (Olympus) and image processing system (Version 1, Leica Imaging Systems Ltd). Ten well-oriented and intact crypt–villus units of each intestinal cross-section were selected for measurements. The villus height/crypt depth ratio was calculated.

### Relative Quantitative Real-Time PCR

Relative messenger RNA (mRNA) abundance of *occludin, ZO-1, claudin-1, interleukin 1*β (*IL-1*β), tumor necrosis factor a (*TNF-*α), interleukin 6 (*IL-6*), and interleukin 10 (*IL-10*) in jejunal and ileal mucosa was determined by real-time PCR as described previously by Wan (34). Briefly, total RNA was extracted using RNAiso Plus (Takara, China) following the manufacturer's guidelines. The RNA samples were reversely transcribed into complementary DNA using RT Reagents (TaKaRa, China) according to the manufacturer's instructions. The primers are listed in Table 2. Quantitative real-time RT-PCR was performed on a PIKO-RT 96 Real-Time PCR Detection System (Thermo Fisher Scientific, America) using SYBR Premix Ex Taq reagent (TaKaRa, China) according to the kit's instructions. Glyceraldehyde-3-phosphate dehydrogenase (GAPDH) was chosen as the reference gene transcript to correct the variances in target gene transcript levels. The reaction was performed in a 10-μl system containing 5 μ SYBR® *Premix Ex* Taq^TM^II (Tli RNaseH Plus, 2 ×), 1 μl RT products, 2 μl double distilled water (ddH_2_O), and 1 μl each of forward and reverse primers at the conditions of 95°C for 7 s followed by 40 cycles of 95°C for 5 s and 60°C for 30 s and a final dissociation step from 60 to 95°C at a heating rate of 0.2°C/s. The 2^−ΔΔCT^ method was used to analyze the relative quantification of gene expression (fold changes), calculated relative to the control group as previously described (23).

**Table 2:** Sequences of primers used for real-time PCR.

**Target**	**Accession number**	**Sense and Antisense primer**	**PCR product (bp)**
GAPDH	AF017079	F:5′GAAGGTCGGAGTGAACGGAT3′	149
		R:5′CATGGGTAGAATCATACTGGAACA3′	
Occludin	NM_001163647.1	F:5′ATGCTTTCTCAGCCAGCGTA3′	176
		R:5′AAGGTTCCATAGCCTCGGTC3′	
ZO-1	XM003353439.1	F:5′GAGGATGGTCACACCGTGGT3′	169
		R:5′GGAGGATGCTGTTGTCTCGG3′	
Claudin-1	NM_001161635.1	F:5′GGACTAATAGCCATCTTTGT3′	88
		R:5′CAGCCATCCGCATCTTCT3′	
IL-1β	NM_214055.1	F:5′ACCTGGACCTTGGTTCTC3′	124
		R:5′GGATTCTTCATCGGCTTC3′	
TNF-α	NM_214022.1	F:5′ACGCTCTTCTGCCTACTGC3′	162
		R:5′TCCCTCGGCTTTGACATT3′	
IL-10	NM_214041.1	F:5′CACTGCTCTATTGCCTGATCTTCC3′	136
		R:5′AAACTCTTCACTGGGCCGAAG3′	
IL-6	NM_214399.1	F:5′TTCAGTCCAGTCGCCTTCT3′	91
		R:5′GTGGCATCACCTTTGGCATCTTCTT3′	

### Serum and Intestinal Mucosa Antioxidant Variables Analysis

Equal amounts of jejunum and ileum mucosa from the same piglet were blended to form a single sample before testing. Antioxidant indexes, including SOD, GSH-Px, T-AOC, and MDA in serum and small intestine mucosa samples were determined using assay kits according to the manufacturer's instructions (Nanjing Jiancheng Bioengineering Institute, Nanjing, China).

### Statistical Analysis

Data were analyzed mean by one-way analysis of variance (ANOVA) using SPSS for windows version 18.0 (SPSS, Inc., Chicago, IL, USA). Differences among treatments were detected by Duncan's multiple range tests. The data are presented as mean, and standard error of the mean (SEM) was given. The *P*-value for significance was set at *P* < 0.05.

## Results

### Growth Performance

The results of growth performance are given in Table 3. Supplementation of the basal diet with CUR + PIP or high-CUR had no effect on the initial weight, final weight, ADFI, and ADG (*P* > 0.05), but the F/G ratio of pigs in the CUR + PIP and high-CUR groups was lower than that in the CON, PIP, and low-CUR groups (*P* < 0.05).

**Table 3:** Effect of curcumin (CUR) and piperine (PIP) on growth performance of weaned Wuzhishan piglets.

**Items**	**Treatments^a^**					**SEM^b^**	***P***
	**CON**	**PIP**	**Low-CUR**	**PIP + CUR**	**High-CUR**		
Initial weight (kg)	3.42	3.66	3.52	3.63	3.49	0.072	0.864
Final weight (kg)	5.01	5.31	5.16	5.62	5.57	0.124	0.535
ADG (g/day)^c^	75.7 2	78.73	78.23	94.77	98.87	3.841	0.171
ADFI (g/day)^d^	172.63	166.80	170.07	175.27	185.83	6.132	0.923
F/G^e^	2.29^f^	2.13^f^	2.18^f^	1.83^g^	1.89^g^	0.054	0.005

a *CON: a corn–soybean basal diet; PIP, low-CUR, PIP + CUR and high-CUR, the basal diet supplemented with 50 mg/kg piperine, 200 mg/kg curcumin, 200 mg/kg curcumin + 50 mg/kg piperine, and 300 mg/kg curcumin, respectively*.

b *SEM, standard error of the mean (n = 10)*.

c *ADG, average daily body weight gain*.

d *ADFI, average daily feed intake*.

e *F/G, the ratio of feed to gain*.

fg *means in the same row with different letters differ significantly (P < 0.05)*.

### Intestinal Mucosa Morphology and Plasma D-Lactate and DAO

The data for intestinal morphology of piglets are shown in Table 4. The weaned piglets fed with CUR + PIP or high-CUR had significantly higher villus height and villus height/crypt depth ratio in jejunal and ileum mucosa than the CON and PIP pigs (*P* < 0.05). The villus height/crypt depth ratio in jejunal and ileum mucosa of pigs in the low-CUR group was higher than that in the CON group (*P* < 0.05), but the villus height was not significantly different between the two groups (*P* > 0.05). The crypt depth and villus width were not significantly different among the five treatments (*P* > 0.05).

**Table 4:** Effect of curcumin (CUR) and piperine (PIP) on intestinal mucosa morphology of weaned Wuzhishan piglets.

**Items**	**Treatments^a^**					**SEM^b^**	***P***
	**CON**	**PIP**	**Low-CUR**	**PIP + CUR**	**High-CUR**		
Jejunum						
Villus height (μm)	314.01^e^	328.50^e^	348.12^de^	377.66^cd^	395.73^c^	9.191	0.003
Villus width (μm)	155.08	140.42	132.51	131.57	133.96	5.389	0.234
Crypt depth (μm)	179.76	176.29	181.28	170.95	173.04	3.941	0.912
Villus height/crypt depth	1.75^e^	1.87^de^	1.92^d^	2.21^c^	2.30^c^	0.062	0.001
Ileum						
Villus height (μm)	298.57^d^	312.63^d^	318.46^d^	354.61^c^	367.86^c^	8.128	0.005
Villus width (μm)	158.23	152.22	159.10	145.11	150.45	3.728	0.805
Crypt depth (μm)	179.97	160.59	151.58	154.76	157.35	3.918	0.458
Villus height/crypt depth	1.71^f^	1.95^e^	2.11^de^	2.29^cd^	2.36^c^	0.069	0.001

a *CON: a corn–soybean basal diet; PIP, low-CUR, PIP + CUR, and high-CUR, the basal diet supplemented with 50 mg/kg piperine, 200 mg/kg curcumin, 200 mg/kg curcumin + 50 mg/kg piperine, and 300 mg/kg curcumin, respectively*.

b *SEM, standard error of the mean (n = 6)*.

cdef *means in the same row with different letters differ significantly (P < 0.05)*.

The plasma d-lactate and DAO results are shown in Table 5. Compared with the control group, the plasma d-lactate and DAO in CUR + PIP and high-CUR groups were significantly lower (*P* < 0.05). Supplementation of PIP significantly reduced plasma DAO activities (*P* < 0.05), but plasma d-lactate was not affected (*P* > 0.05). Moreover, the plasma d-lactate and DAO were significantly different between low-CUR and control groups (*P* > 0.05).

**Table 5:** Effect of curcumin (CUR) and piperine (PIP) on plasma d-lactate and diamine oxidase activity (DAO) in weaned Wuzhishan piglets.

**Items**	**Treatments^a^**					**SEM^b^**	***P***
	**CON**	**PIP**	**Low-CUR**	**PIP + CUR**	**High-CUR**		
Plasma DAO (U/L)	7.17^c^	6.41^d^	6.55^cd^	5.58^e^	5.29^e^	0.199	0.001
Plasma d-lactate (mg/L)	5.91^c^	5.22^c^	5.34^c^	4.23^d^	3.67^d^	0.240	0.001

a *CON: a corn–soybean basal diet; PIP, low-CUR, PIP + CUR, and high-CUR, the basal diet supplemented with 50 mg/kg piperine, 200 mg/kg curcumin, 200 mg/kg curcumin + 50 mg/kg piperine, and 300 mg/kg curcumin, respectively*.

b *SEM, standard error of the mean (n = 6)*.

cde *means in the same row with different letters differ significantly (P < 0.05)*.

### mRNA Expressions of Tight Junction Proteins

**Figure 1:**
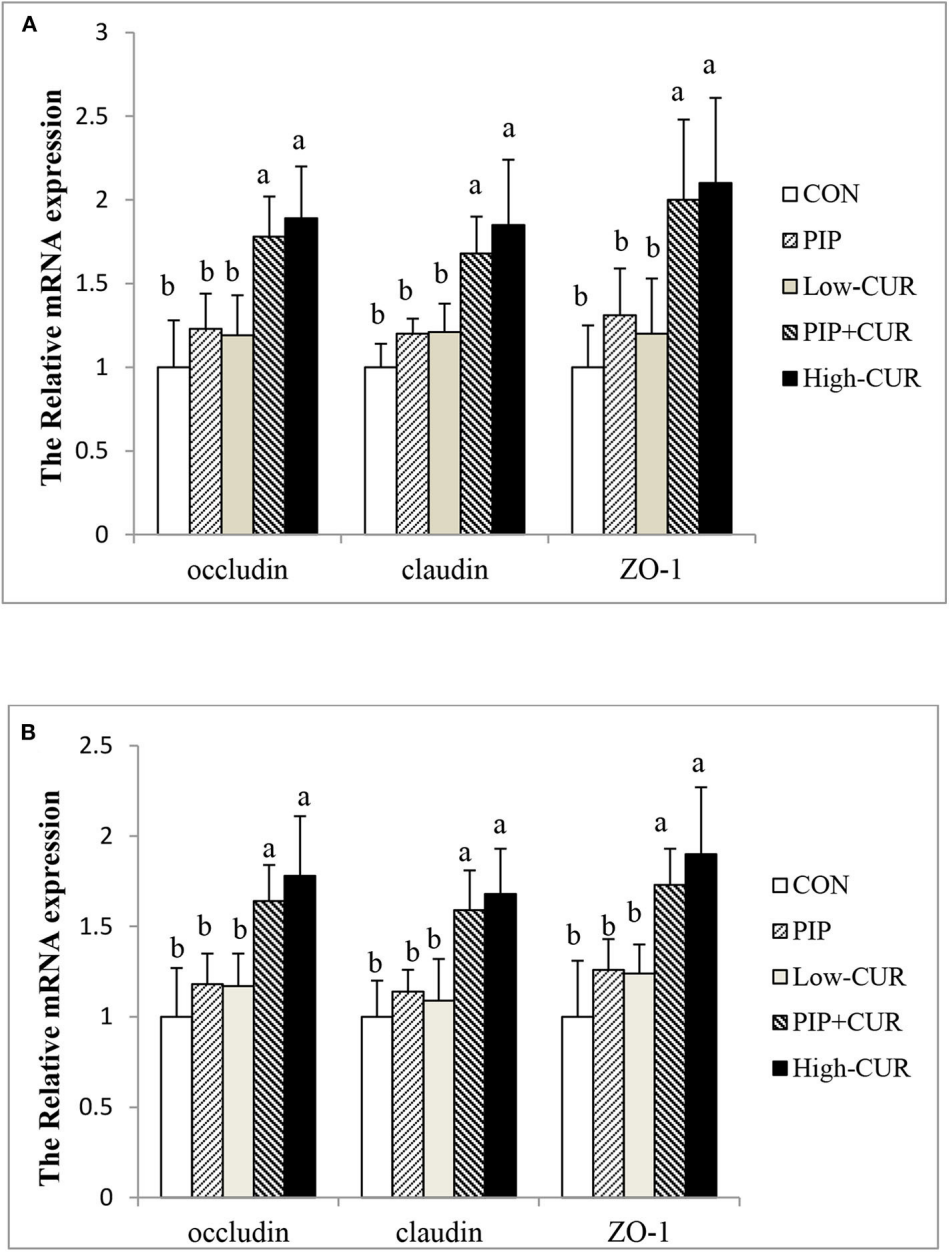
Effect of curcumin (CUR) and piperine (PIP) on messenger RNA (mRNA) expression of tight junction protein in **(A)** jejunal mucosa and **(B)** ileal mucosa of weaned Wuzhishan piglets. Values are expressed as mean ± standard deviations (*n* = 6). ^ab^Means values within different letters differ significantly (*P* < 0.05). CON, a corn–soybean basal diet; PIP, low-CUR, PIP + CUR, and high-CUR, the basal diet supplemented with 50 mg/kg piperine, 200 mg/kg curcumin, 200 mg/kg curcumin + 50 mg/kg piperine, and 300 mg/kg curcumin, respectively.

Figure 1 shows the mRNA expression of *occludin, claudin-1*, and *ZO-1* in the jejunal and ileum mucosa of piglets. The expression levels of *occludin, claudin-1*, and *ZO-1* in the jejunal and ileum mucosa of piglets in the CUR + PIP and high-CUR groups were significantly higher than those in the control, PIP, and low-CUR groups (*P* < 0.05). However, there were no significant differences in the expression of above substances between the PIP and low-CUR groups (*P* > 0.05).

### mRNA Expressions of Cytokines

**Figure 2:**
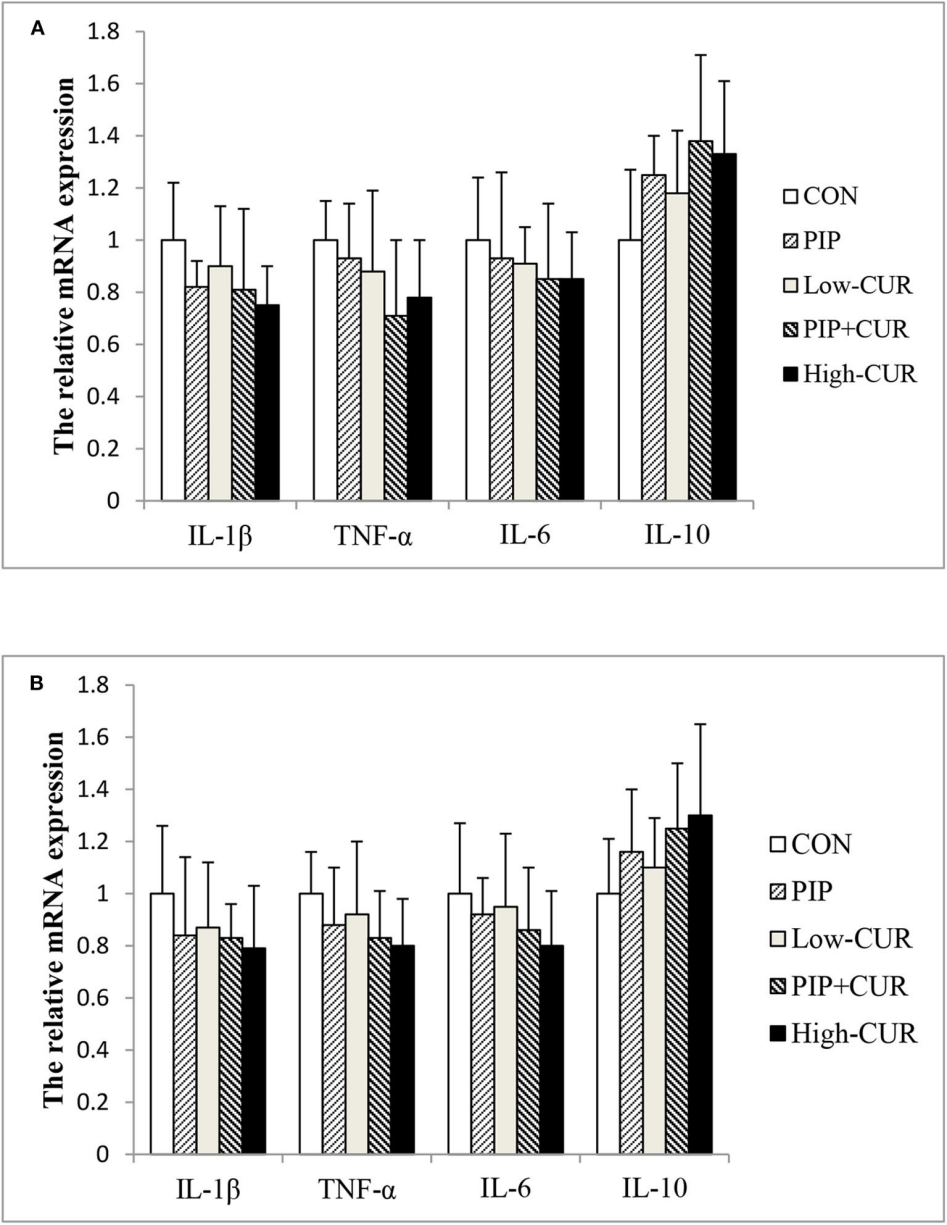
Effect of curcumin (CUR) and piperine (PIP) on messenger RNA (mRNA) expression of cytokines in **(A)** jejunal and **(B)** ileal mucosa of weaned Wuzhishan piglets. Values are means (*n* = 6), and standard deviations represented by vertical bars. CON, a corn–soybean basal diet; PIP, low-CUR, PIP + CUR, and high-CUR, the basal diet supplemented with 50 mg/kg piperine, 200 mg/kg curcumin, 200 mg/kg curcumin + 50 mg/kg piperine, and 300 mg/kg curcumin, respectively. Dietary Rice Improves Growth Performance, Mucosal Enzyme Activities, and Plasma Urea Nitrogen in Weaning Piglets. Effects of Dietary Methionine Supplementation on Growth Performance, Intestinal Morphology, Antioxidant Capacity, and Immune Function in Intra-Uterine Growth-Retarded Suckling Piglets. Comparative Effects of Dietary Supplementations With Sodium Butyrate, Medium-Chain Fatty Acids, and n-3 Polyunsaturated Fatty Acids in Late Pregnancy, and Lactation on the Reproductive Performance of Sows and Growth Performance of Suckling Piglets. Effects of Composite Antimicrobial Peptide on Growth Performance and Health in Weaned Piglets.

Figure 2 shows the mRNA expression of cytokines in the jejunal and ileum mucosa of piglets. The mRNA levels of *IL-1*β, *TNF-*α, *IL-6*, and *IL-10* were not significantly different among the five treatment groups (*P* > 0.05).

### Serum and Intestinal Mucosa Antioxidant Parameters

As shown in Table 6, pigs in CUR + PIP and high-CUR groups had higher SOD and GSH-Px activities as well as lower MDA concentration in serum and intestinal mucosa than pigs in the CON group (*P* < 0.05). The PIP piglets had higher GSH-Px activity and lower MDA concentration in the serum and intestinal mucosa than the CON piglets (*P* < 0.05). The value of T-AOC in serum and intestinal mucosa was not significantly different among the five treatments (*P* < 0.05).

**Table 6:** Effect of curcumin (CUR) and piperine (PIP) on antioxidant variables in serum and intestinal mucosa of weaned Wuzhishan piglets.

**Items**	**Treatments^a^**					**SEM^b^**	***P***
	**CON**	**PIP**	**Low-CUR**	**PIP + CUR**	**High-CUR**		
Serum (U/mL)							
Superoxide dismutase	114.57^e^	129.89^de^	122.80^de^	147.24^cd^	167.24^c^	8.40	0.019
Total antioxidant capacity	1.22	1.27	1.36	1.37	1.42	0.103	0.475
Glutathione peroxidase	160.73^e^	234.11^cd^	205.98^d^	258.61^c^	272.60^c^	13.17	0.001
Malondialdehyde (nmol/ml)	5.57^c^	4.70^de^	5.16^cd^	4.12^e^	4.05^e^	0.18	0.003
Intestinal mucosa (U/mg protein)							
Superoxide dismutase	4.16^d^	4.69^d^	4.45^d^	5.74^c^	5.52^c^	0.179	0.005
Total antioxidant capacity	0.54	0.64	057	0.64	0.73	0.035	0.239
Glutathione peroxidase	20.44^e^	36.32^d^	29.50^de^	44.40^c^	48.47^c^	2.958	0.015
Malondialdehyde (nmol/mg protein)	0.49^c^	0.35^d^	0.36^d^	0.24^e^	0.22^e^	0.027	0.001

a *CON: a corn–soybean basal diet; PIP, low-CUR, PIP + CUR, and high-CUR, the basal diet supplemented with 50 mg/kg piperine, 200 mg/kg curcumin, 200 mg/kg curcumin + 50 mg/kg piperine, and 300 mg/kg curcumin, respectively*.

b *SEM, standard error of the mean (n = 6)*.

cde *means in the same row with different letters differ significantly (P < 0.05)*.

## Discussion

In recent years, there have been several attempts to demonstrate the use of CUR as a potential feed additive that can replace antimicrobial growth promoters. Diets supplied with 50 and 100 mg/kg CUR increased the growth performance of broilers by improving the antioxidant defense system and enhancing the mitochondrial biogenesis. Ruan et al. (20) found that CUR prevented the decrease in body weight and ADG in ducks fed with corn contaminated by ochratoxin A. Ilsley et al. (35) reported that dietary supplementation with 200 mg/kg CUR had no influence on pig growth performance. This finding was in line with that of our previous study, which suggested that no growth improvement was observed in weaned pigs fed with 200 mg/kg CUR-added diet (23). In the present experiment, supplementation of CUR + PIP or high-CUR significantly improved the growth performance of pigs by reducing F/G, indicating that high-CUR or CUR + PIP had better performance-promoting effects than PIP or low-CUR added alone in piglet diets.

As common indicators for estimating intestinal integrity, the villus height, crypt depth, villus width, and the villus height/crypt depth ratio can reveal some information on gut health in pigs. Increasing the villus height suggest an increased surface area for nutrient absorption (36). The villus crypt is considered as villus factory. The increase in crypt depth indicates fast tissue turnover and high demand for new tissue, which are generally associated with decline in nutrient digestion and absorption capacity (37, 38). Studies have confirmed that weaning is associated with villus atrophy and crypt hyperplasia (3, 39). In the present study, the increase in villus height and villus height/crypt depth ratio in jejunal and ileum mucosa caused by the addition of CUR + PIP or high-CUR was observed. Similar results were achieved in our previous study, which noticed that the addition of 400 mg/kg CUR in the diet increased the villus height and villus height/crypt depth ratio in piglets, demonstrating that both the supplementation with CUR + PIP and the addition of high-CUR could ameliorate the weaning-associated damage to small intestinal morphology, thus correspondingly improving the digestion and absorption of nutrients and promoting the growth performance (23).

The integrity of intestinal mucosa barrier is the basis for the normal function of epithelial cells and defense against the pathogenic bacteria (3). Plasma DAO and d-lactate are used as sensitive circulating indicators of the severity of mucosal injury (40, 41). DAO exists only in the villi of the upper small intestine, and a small amount is normally present in the blood. When the intestinal mucosal function is injured, mucosal permeability increases, promoting the release of more endocellular DAO into the blood (42). Therefore, plasma DAO reflects the integrity of intestinal mucosa. d-Lactate is the end product of intestinal bacterial fermentation. When the intestinal mucosal function is impaired, the d-lactate concentration in blood is increased, which is because d-lactate in mammals cannot be metabolized due to the lack of enzyme systems (43). Studies have shown that early weaning leads to impaired mucosal barrier function and increased intestinal permeability (2, 44). The present results showed that dietary supplementation of CUR + PIP or high-CUR improved intestinal barrier function by reducing plasma DAO and d-lactate. The data were supported by our previous studies, which revealed that dietary addition of 300 or 400 mg/kg CUR reduced plasma DAO and d-lactate in weaned piglets challenged with enterotoxigenic *E. coli* (23).

Tight junction proteins (*occludin, claudin-1*, and *ZO-1*) play an important role in the maintenance of intestinal mucosal barrier integrity. They function as the continuous intercellular barrier against the translocation of intestinal bacteria, antigens, and intraluminal toxins from the lumen into subepithelial tissue and systemic blood circulation (45). It has been established by several studies that CUR promotes the expression of tight junction proteins in intestinal mucosa. Tian et al. (46) found that CUR significantly upregulated the expression of *ZO-1* following the intestinal ischemia–reperfusion injury in rats, which might be partly attributed to the TNF-α related pathway. The study of Ruan et al. (20) implied that CUR increased jejunal mucosa occludin and *ZO-1* mRNA and protein levels in ducks. In the present study, the upregulation of *occludin, claudin-1*, and *ZO-1* mRNA expression due to the supplementation with CUR + PIP or high-CUR suggested that both treatments might improve the intestinal integrity. The results were consistent with the improved intestinal morphology and decrease in the plasma d-lactate and DAO levels. The molecular mechanism of CUR + PIP in regulation of tight junctions requires further study.

In addition to intestinal integrity, weaning-associated intestinal inflammation was also observed in weaning piglets (39, 47). Weaning causes the upregulation of proinflammatory cytokines, such as *TNF-*α, *IFN-*γ, *IL-1*β, and *IL-6*. Overproduction of proinflammatory cytokines induces a pathological opening of the intestinal tight junctions and increases intestinal epithelial permeability, resulting in intestinal barrier dysfunction (48, 49). Song et al. (19) noticed that CUR decreased the mRNA expression of *IL-1*β and *TNF-a* and increased the mRNA expression of *IL-10* in intestinal mucosa of rats and IEC-6 cells. Ruan et al. (20) also observed that CUR decreased the concentrations of *TNF-*α and *IL-1*β induced by OTA in jejunal mucosa of ducks. In our previous studies, the mRNA levels of *TNF-*α, *IL-6*, and *IL-1*β were decreased by supplementation with 400 mg/kg CUR in weaned piglets challenged with enterotoxigenic *E. coli*. However, in the present experiment, the mRNA expression levels of *IL-1*β, *TNF-*α, *IL-6*, and *IL-10* were not affected by dietary supplementation. The present study results demonstrated that the transiently upregulated inflammatory cytokines induced by weaning could rapidly return to the preweaning level 9 days after weaning, which might be the reason why dietary CUR supplementation did not affect mRNA expression of inflammatory cytokines 21 days after weaning.

Studies have confirmed that weaning induces oxidative stress and increases free radicals in tissue and blood. Antioxidants can eliminate free radicals and reduce oxidative stress. CUR has the potential *in vivo* antioxidant activity owing to its ability to scavenge reactive oxygen species (50, 51) and inhibit lipid peroxidation (52). The gastrointestinal tract can remove free radicals and prevent oxidative damage, mainly serving as antioxidant in the human body. Antioxidant enzymes include GSH-Px, SOD, CAT, and MDA (52). MDA is the end product of lipoperoxidation, and the levels of MDA in plasma and tissue are an excellent oxidative stress marker (53). In our study, both CUR + PIP and high-CUR increased the activities of serum and intestinal mucosa antioxidant enzymes (e.g., GSH-Px and SOD) and decreased the lipid peroxidation marker MDA, indicating that the oxidative stress was reduced by dietary CUR or CUR + PIP supplementation. This result was similar with that of the study of Arcaro et al. (54) who reported that the level of MDA was markedly reduced in the plasma of diabetic rats fed with 90 mg/kg curcumin or 90 mg/kg CUR + 20 mg/kg PIP. Oxidative stress caused by weaning is responsible for intestinal mucosal injury (55). Reduced oxidative stress might alleviate the damage of intestinal mucosal barrier, and high-CUR or CUR + PIP supplementation decreased the intestinal permeability subsequently.

## Conclusions

In summary, the present study showed that supplementation with PIP + CUR or high-CUR could reduce intestinal permeability, enhance antioxidant capacity, and had beneficial effects on feed utilization rate of the corn–soybean basal diet. The addition of both CUR and PIP appeared to be as advantageous as high-CUR, but it was more effective than low-CUR or PIP alone.

## Data Availability Statement

All datasets presented in this study are included in the article/supplementary material.

## Ethics Statement

The animal study was reviewed and approved by Committee on laboratory animal ethics of Tropical Crops Genetic Resources Institute (TCGRI). Written informed consent was obtained from the owners for the participation of their animals in this study.

## Author Contributions

LS and WX designed the experiments. TC, WP, and HH carried out the feeding experiments. GH analyzed the experimental results. All authors contributed to the article and approved the submitted version.

## Conflict of Interest

The authors declare that the research was conducted in the absence of any commercial or financial relationships that could be construed as a potential conflict of interest.
